# Primary Intracranial Hemorrhage: Characteristics, Distribution, Risk Factors, and Outcomes—A Comparative Study between Jewish and Arab Ethnic Groups in Northern Israel

**DOI:** 10.3390/jcm12154993

**Published:** 2023-07-29

**Authors:** Jamal Saad, Chen Hanna Ryder, Mahmod Hasan, Galina Keigler, Samih Badarny

**Affiliations:** 1 Azrieli Faculty of Medicine, Bar Ilan University, Safed 1311502, Israel; 2 Brain & Behavior Research Institute, Western Galilee Academic College, Acre 2412101, Israel; 3 Orthopedic Department, Galilee Medical Center, Nahariya 2210001, Israel; 4 Department of Neurology, Galilee Medical Center, Nahariya 2210001, Israel

**Keywords:** intracerebral hemorrhage (ICH), stroke, risk factors, hemorrhage volume, functional outcome, ethnic differences, Jewish, Arab

## Abstract

**Background and purpose:** This study aimed to investigate the differences in intracerebral hemorrhage (ICH) between Jews and Arabs residing in northern Israel, focusing on risk factors, hemorrhage volume, and functional outcome. **Methods:** A retrospective analysis was conducted utilizing a population-based registry to investigate intracerebral hemorrhage (ICH) characteristics, risk factors, and outcomes. The registry consisted of inpatients diagnosed with hemorrhagic stroke. Due to the wide variation in data on ICH characteristics and the limited availability of population-based data on predictors of ICH survival and functional outcomes, we collected retrospective data on all adult patients admitted to the Galilee Medical Center with a diagnosis of ICH. Data were obtained from the registry covering the period from 2013 to 2019. Ethnic differences and risk factors associated with intracranial hemorrhage (ICH) were examined within a diverse population of 241 patients, comprising 52.70% Jews (*n* = 127) and 47.30% Arabs (*n* = 114). **Results:** The results of this study revealed significant differences in age, obesity rates, and intracerebral hemorrhage (ICH) location between the two ethnic groups. Hypertension emerged as the most prevalent condition among ICH patients in both ethnic groups (76.70%), followed primarily by anticoagulant use (63.60%), dyslipidemia (60.70%), diabetes (44.60%), obesity (30.60%), smoking (24.60%), and a history of cardiovascular disease (21.80%). Furthermore, 20.90% of the patients had a history of previous cerebrovascular accidents (CVA). Arab patients with ICH were generally younger (62.90 ± 16.00 years) and exhibited higher rates of obesity (38.70%) compared to Jewish patients with ICH (70.17 ± 15.24 years, 23% obesity; *p* = 0.001, *p* = 0.013, respectively). Hemorrhage volume was identified as a crucial determinant of patient outcomes, with larger volumes associated with poorer Modified Rankin Scale (mRS) scores at discharge and higher mortality rates. Interestingly, patients without hypertension had higher hemorrhage volumes compared to those with hypertension. The extent of hemorrhage into the ventricles did not significantly correlate with mRS at discharge in our dataset. **Conclusions:** This study highlights significant differences in the characteristics and outcomes of intracranial hemorrhage (ICH) between Jews and Arabs in northern Israel. The findings reveal variations in age, obesity rates, and ICH location between the two groups. While hypertension was the most prevalent risk factor for both populations, other risk factors differed. Notably, hemorrhage volume emerged as a crucial prognostic factor, aligning with previously published data. These findings underscore the necessity for tailored approaches that consider ethnic-specific factors in the risk assessment, prevention, and management of ICH. Further research is warranted to elucidate the underlying mechanisms and develop interventions aimed at improving outcomes and enhancing healthcare practices in ICH management.

## 1. Introduction

Intracerebral hemorrhage (ICH) is a neurological condition characterized by bleeding within the brain, resulting in disrupted brain function [1]. It accounts for 10–30% of stroke admissions and carries a 30–50% mortality rate within six months [2]. Risk factors include hypertension, smoking, excessive alcohol consumption, dyslipidemia, obesity, the use of anticoagulant and antithrombotic agents, old age, male sex, cerebral amyloid angiopathy (CAA), Asian ethnicity, diabetes, chronic atrial fibrillation, and a history of cardiovascular disease [1,3,4,5,6]. Early detection and management of these risk factors are crucial. The treatment in ICH aims to control bleeding, reduce brain swelling, and manage associated conditions [7,8]. Multidisciplinary rehabilitation and long-term follow-up care are essential for preventing recurrence and managing outcomes [9].

Approximately half of ICH cases originate in the basal ganglia, one-third in the cerebral hemispheres, and one-sixth in the brainstem or cerebellum [7]. Intraventricular hemorrhage accompanies intracerebral hemorrhage (ICH) in 40% of cases, leading to acute hydrocephalus and elevated intracranial pressure (ICP). Without timely medical intervention, these factors may significantly worsen the prognosis [10].

Understanding the risk factors, pathophysiology, and management of ICH is crucial for improving patient outcomes and reducing the burden of this devastating condition. The National Institutes of Health Stroke Scale (NIHSS) was initially designed as a research tool to measure baseline data in acute stroke clinical trials. However, it is now widely used as a clinical assessment tool to evaluate stroke severity, determine appropriate treatment, and predict patient outcomes. In primary ICH, the NIHSS may serve as a better predictor of outcome than the Glasgow Coma Scale (GCS) alone, as demonstrated in various cohorts of ICH patients through multivariate analyses [11].

Israel’s diverse population comprises three primary ethnic groups, each contributing to the nation’s rich social fabric. Jews constitute the majority at 74%, followed by Arabs representing 21.1% (including Muslims, Druses, Circassians, and a minority of Christian Arabs), and the remaining 4.9% comprises other groups such as non-Arab Christians and citizens with no religious affiliation. These ethnic groups exhibit distinct genetic, demographic, and cultural characteristics, as well as varied manifestations of nutritional and health lifestyles [12,13,14,15]. However, there are limited data available concerning the incidence of intracerebral hemorrhage (ICH) among these prevalent ethnic groups [13]. This study seeks to address this knowledge gap by investigating potential differences in risk factors, characteristics, and outcomes of ICH between the Jewish and Arab populations residing in northern Israel. Additionally, the current study will investigate the role of hemorrhage volume as a prognostic factor for patient outcomes, utilizing Modified Rankin Scale (mRS) scores at discharge [13].

Previous research has suggested that the two main ethnic populations in Israel differ in the causes and attributes of ICH, with specific associations of heavy smoking and poorly controlled diabetes in the Arab population [13]. Differences in health status, morbidity, mortality, and risk factors for non-communicable diseases have also been observed between these subpopulation groups [14]. Levels of stroke knowledge among Arab-Muslim Israelis were reported to be low to moderate, indicating the need for targeted interventions [16].

Dietary patterns show significant ethnic differences, highlighting the necessity for culturally congruent dietary interventions to address nutrition-related chronic disease differences between Arabs and Jews [12]. Understanding the distinctions in ICH characteristics, risk factors, and outcomes between these two ethnic groups is crucial for guiding healthcare strategies and policies, potentially leading to improved health outcomes in diverse populations while enhancing our understanding and management of ICH.

## 2. Materials and Methods

### 2.1. Study Design 

This retrospective population-based registry study included adult patients (aged 18 years and above) diagnosed with intracranial hemorrhage (ICH) and admitted to the Galilee Medical Center between 1 January 2013, and 31 December 2019. Exclusion criteria comprised patients under 18 years old or those with secondary ICH due to trauma, neoplasm, vascular malformation, or ischemic stroke that transformed into hemorrhagic stroke. Trained personnel collected the data.

### 2.2. Data Collection 

Demographic and clinical data were collected, including age, sex, comorbidities (hypertension, diabetes, obesity (BMI > 30), dyslipidemia, atrial fibrillation, ischemic heart disease), drug use (anticoagulation), hemostasis parameters (PT INR, aPTT), smoking status, stroke subtype, stroke severity, and functional outcome at discharge. Stroke subtype was classified based on the hemorrhage location: “Deep Regions” referred to hematomas in the basal ganglia or thalamic regions, “Posterior Fossa” to hematomas in the cerebellum and brainstem, and “Lobar Regions” to hematomas in the frontal, temporal, and occipital lobes. Stroke severity was assessed using a combination of National Institutes of Health Stroke Scale (NIHSS), hemorrhage volume, intraventricular hemorrhage, midline shift, and surgical intervention. In-hospital mortality and functional outcomes were recorded.

Functional status at discharge was evaluated using the Modified Rankin Scale (mRS). Discharge locations included home, nursing homes, non-neurological in-hospital departments, and out-hospital rehabilitation centers.

### 2.3. Calculation of Hemorrhage Volume

The volume of ICH was calculated using the ABC/2 formula, which involves measuring the length, width, and height of the hemorrhage on a CT scan and applying a mathematical formula to determine the volume in cubic centimeters (cc). The formula used was A × B × C/2, where A is the greatest hemorrhage diameter by CT, B is the diameter 90° to A, and C is the approximate number of CT slices with hemorrhage multiplied by the slice thickness. This method has been validated in previous studies and is commonly used in clinical practice to assess the volume of intracerebral hemorrhage [17,18].

## 3. Statistical Analysis 

### 3.1. Descriptive Analysis

Categorical data were described using frequencies and percentages. Continuous variables with a normal distribution were presented as mean ± standard deviation, while variables that did not meet the normal distribution assumption were presented as median value and range.

### 3.2. Inferential Analysis

Categorical variables were compared between groups using the Chi-square test, or, alternatively, Fisher’s exact tests when the expected count was less than 5. Continuous variables were compared between groups using the Independent *t*-test or Mann–Whitney test based on variable distribution. An independent *t*-test was used if a normal distribution was found, and the decision regarding the distribution shape was determined by a histogram. A significance level of *p* < 0.05 was considered statistically significant.

The statistical analysis was performed using Statistical Product and Service Solutions (SPSS) Version 27.0 software.

## 4. Ethical Considerations 

This retrospective observational–analytical study involved no intervention. The study design and data collection were approved by the Galilee Medical Center’s local Helsinki Committee. As the study involved a retrospective analysis of anonymized patient data, the need for informed consent was waived. All data were handled following relevant guidelines and regulations to ensure patient privacy and confidentiality.

## 5. Results

The study included a total of 241 patients with intracranial hemorrhage (ICH), comprising 127 (52.70%) Jews and 114 (47.30%) Arabs. The mean age for the two groups was 66.77 ± 15.84 years, and the majority of patients were males (61.80%). Arab ICH patients were relatively younger (62.90 ± 16.00 years) and exhibited higher rates of obesity (38.70%) compared to Jewish ICH patients (70.17 ± 15.24 years, 23% obesity; *p* = 0.001, *p* = 0.013, respectively) (Table 1).

The most prevalent risk factors for intracranial hemorrhage among the patients included hypertension (76.70%), anticoagulant use (63.60%), dyslipidemia (60.70%), diabetes (44.60%), obesity (30.60%), a history of cardiovascular disease (21.80%), and smoking (24.60%). Furthermore, 20.90% of the patients had a history of previous cerebrovascular accidents (CVA) (Table 1).

Upon admission, a total of 75.30% of patients achieved a good (0–2) mRS score, with a median NIHSS of 6.00 ± 5.80 and a median Glasgow Score of 15.00 ± 3.84. Only one third of the patients were discharged to their homes (33.20%). The mean systolic and diastolic pressures upon admission were 156.84 ± 32.30 and 84.63 ± 18.95, respectively (Table 2).

The median hemorrhage volume was 5.10 ± 17.92 cc. A significant difference was observed in the hemorrhage volume between patients who survived hospitalization and those who did not (*p* < 0.001), as well as between patients discharged to their homes and those discharged elsewhere (*p* < 0.001). The latter group demonstrated a higher volume of hemorrhage. Surprisingly, no significant difference was found in the hemorrhage volume between patients with good and poor mRS scores upon admission (*p* = 0.504). However, a significant difference was observed in the hemorrhage volume between patients with good and poor mRS scores at discharge (*p* = 0.001), with the poor mRS group having higher volumes. Interestingly, patients without hypertension had higher hemorrhage volumes. The median hemorrhage volume for patients with hypertension was 4.4 cc [0.10–103.20], while, for patients without hypertension, it was 8.95 cc [0.10–93.20]. Additionally, a significant statistical difference was found in surgical intervention between patients with a poor Modified Rankin Scale (mRS) score at discharge and those with a good mRS score at discharge, as demonstrated by the Chi-square test two-sided *p* = 0.005. The percentage of patients with a poor mRS who needed surgical intervention is higher compared to patients with a high mRS at discharge.

The platelet counts were significantly higher in the Arab patient population (239.08 ± 77.98 vs. 215.89 ± 63.93, *p* = 0.013) (Table 2).

The primary differences between the two ethnic groups were observed in the location of the intracranial hemorrhage (ICH) (*p* = 0.007). Arab ICH patients had a higher rate of posterior fossa ICH (20.70%) compared to Jewish ICH patients (8.70%; *p* = 0.007). Conversely, Jewish ICH patients exhibited a higher prevalence of lobar ICH (35.40%) compared to Arab ICH patients (21.60%). However, the deep regions were the most common location of hemorrhage for both groups, with 135 cases (56.70%) in total: 71 (55.90%) among Jews and 64 (57.70%) among Arabs. Furthermore, the hemorrhage volume in the lobar regions group was larger compared to the deep regions and posterior fossa, while the hemorrhage volume in the deep regions group was smaller in comparison to the other two location groups (*p* < 0.001) (Table 3).

In terms of ICH location, deep regions were identified as the most prevalent (56%). Table 4 provides insights into the age differences between Arabs and Jews in each hemorrhagic region. The results indicate that Arab patients were significantly younger than Jewish patients in both the deep regions (64.10 ± 15.10 vs. 71.20 ± 14.10, *p* = 0.005) and lobar regions (61.20 ± 17.90 vs. 69.70 ± 17.20, one-sided *p* = 0.029), as well as in the total population (Table 4 and Table 1).

## 6. Discussion

This study provides valuable insights into ethnic differences and associated risk factors in intracranial hemorrhage (ICH) within a diverse patient cohort. The results highlight significant differences in ICH characteristics between Arab and Jewish patients, including age, obesity rates, and hemorrhage location. Additionally, the results emphasize the importance of hemorrhage volume as a crucial factor affecting patient outcomes, consistent with previous studies’ findings [19,20]. When interpreting the results of this study, it is essential to recognize that our analysis was exploratory in nature. Our primary goal was to maintain the original sample with an exclusive division based on ethnicity, which led us to refrain from adjusting for potential confounders such as sex and comorbidities. Consequently, the associations observed between various factors and outcomes should be approached with caution, as they do not fully account for the potential influence of these confounding variables. In the current study, Arab ICH patients were generally younger and had higher obesity rates compared to their Jewish counterparts, in line with other studies in Israel [21]. These observations may be attributed to pathological lifestyle factors, as supported by previous research on Arab patients in Israel [13,22,23,24]. These studies have consistently reported a higher frequency of modifiable risk factors such as diabetes and smoking among Arab patients, which could help explain the differences in ICH characteristics between Arab and Jewish populations in our study. Such factors play a critical role in the pathogenesis of cerebral small vessel arteriopathy and, subsequently, ICH, particularly in the deep regions [25]. Compliance and adherence to treatment recommendations may also contribute to the observed differences.

The observed trends also align with a multicenter study on cerebral hemorrhage in Italy, which reported a significant indirect effect of obesity on the risk of deep ICH [26]. This similarity further supports the notion that obesity may play a crucial role in the development of specific types of ICH and reinforces the importance of addressing modifiable risk factors in stroke prevention strategies.

Regarding the results that Jewish patients tended to be older than their Arab counterparts, one possible explanation may be related to cerebral amyloid angiopathy (CAA), which is known to have a strong correlation with age [27] and primarily affects small blood vessels within cortical and subcortical regions of the brain [28]. Consequently, hemorrhages associated with CAA are often situated superficially in the brain. This age-related predisposition for CAA-related hemorrhages could possibly contribute to the higher incidence of lobar ICH observed within the Jewish demographic.

Another finding in our study was that hypertension emerged as the most prevalent condition among ICH patients in both ethnic groups, followed by anticoagulant use, hyperlipidemia, diabetes, obesity, and smoking. These findings align with existing literature on ICH risk factors and underscore the importance of early detection and management of modifiable risk factors to prevent ICH and its complications.

Interestingly, a high proportion of patients presenting with mild clinical symptoms in both ethnic groups was observed. This observation might be attributable to several factors: the initial severity of symptoms in ICH can be influenced by the location and size of the hemorrhage, the presence of comorbidities, and the overall health status of the patient [29]. As such, it is not entirely unexpected to observe a significant proportion of patients presenting with milder symptoms.

Furthermore, it is important to note that severe ICH can be rapidly fatal [30], potentially leading to an overrepresentation of milder cases in a hospital setting, as these patients have a higher likelihood of surviving until hospital admission. Improved public awareness about the signs and symptoms of stroke could have also played a role, leading to early hospital presentation and thereby increasing the proportion of documented mild cases.

Despite the initial mild presentation, it is noteworthy that the need for further care was significant, and the rate of discharge to home was relatively low. This indicates that these events, though initially presenting as mild, had substantial impacts on patients’ health status and required comprehensive management and rehabilitation.

Our study also revealed the crucial role of hemorrhage volume in determining patient outcomes. Larger volumes were associated with poorer Modified Rankin Scale (mRS) scores at discharge and increased mortality rates [31,32]. This finding highlights the need for early intervention strategies aimed at minimizing hemorrhage volume to improve patient outcomes. Notably, patients without hypertension had higher hemorrhage volumes compared to those with hypertension. The deep regions were identified as the most likely location for ICH associated with hypertension, and the hemorrhage volume in the deep regions was smaller compared to other regions, consistent with previous studies indicating that lobar hemorrhage tends to have a larger volume than deep regions [31,32]. Further investigation is warranted to explore the underlying mechanisms of ICH development.

In the current study, the Modified Rankin Scale (mRS) at discharge was used as a primary outcome measure. Although the National Institutes of Health Stroke Scale (NIHSS) is a well-established tool for assessing the severity of stroke and has been shown to be a good predictor of outcome in ICH patients [33,34], we chose to use the mRS at discharge for several reasons.

Firstly, while NIHSS is an excellent tool for assessing neurological impairment immediately after stroke, the mRS is a clinically relevant scale, with six different clear and well-defined grades. It is also proven to have acceptable reliability and is correlated with the Barthel Index (BI) [35]. The mRS has few variations in stroke trials [36,37], making it suitable to use mRS cutoff scores as a reference to distinguish between favorable and unfavorable outcomes. Moreover, it evaluates a patient’s ability to carry out daily activities, making it a more comprehensive measure of functional outcomes after ICH.

Secondly, the mRS is widely used in clinical practice and research as an outcome measure in stroke studies, and it is particularly helpful when examining outcomes at discharge or at follow-up time points. Given that our study aimed to explore patient outcomes at the time of discharge, the mRS was deemed to be a more appropriate tool.

However, we acknowledge the importance and utility of NIHSS in predicting outcomes in ICH patients.

In the literature, intraventricular hemorrhage (IVH) has been associated with a worsened prognosis in ICH patients [38,39,40]. However, our study did not find a significant association with mRS at discharge. The lack of a significant association between IVH and mRS at discharge in our study may be attributed to milder IVH severity in our patient group compared to previous studies. Additionally, ventricular extension alone might not strongly predict patient outcomes, warranting consideration of other clinical factors when assessing prognosis.

While this study provides valuable insights into the differences in intracranial hemorrhage (ICH) characteristics and outcomes between Jews and Arabs residing in northern Israel, it is important to acknowledge its limitations. Firstly, the study’s retrospective design may have limitations in the completeness and accuracy of data collection. Some potentially relevant variables may not have been consistently documented in all patients’ medical records, which could introduce biases in the analysis. Secondly, our results are based on a single-center experience, which may restrict the generalization of our findings. The demographics of the patients treated at our center might not fully represent the broader populations of Jews and Arabs. Further research, preferably prospective and multicenter, is needed to explore potential causal factors and underlying mechanisms behind these observed differences.

Accordingly, future studies should consider these factors to provide a more comprehensive understanding of the differences in ICH outcomes. Given the complex interplay of various demographic, clinical, and lifestyle factors in the development and outcomes of ICH, future research could benefit from a larger sample of patients and a more comprehensive analysis that adjusts for potential confounders. This approach could provide a more nuanced understanding of the differences observed in ICH characteristics and outcomes between different ethnic groups and contribute to the development of targeted prevention and management strategies. Multifaceted research efforts will be critical for advancing our knowledge and improving the care of patients with ICH in these populations.

## 7. Conclusions

In conclusion, this study investigated differences in intracranial hemorrhage (ICH) characteristics and outcomes between Jewish and Arab populations in northern Israel. Arab patients, comparatively younger and presenting higher obesity rates than their Jewish counterparts, underscored the significant role of age, metabolic factors, and lifestyle in influencing ICH risk. Hypertension emerged as a prevalent associated factor in both groups, yet the prevalence of other risks varied, underlining the need for ethnicity-specific preventive strategies. In terms of ICH characteristics, Arab patients were more likely to exhibit posterior fossa ICH, while Jewish patients suffered more from lobar ICH. Furthermore, Arab patients demonstrated higher platelet counts. Some of these characteristics were found to be associated with patient outcomes, accentuating their potential influence on prognosis. In light of these findings, future comprehensive, multi-center research is indispensable for facilitating the development of tailored intervention and prevention strategies. A deeper understanding of ethnicity-specific ICH characteristics is a steppingstone towards the design of more effective, targeted management approaches for this severe medical condition.

## Figures and Tables

**Table 1:** Demographics and risk factors of ICH patients.

Characteristics	Jews *n* = 127	Arabs *n* = 114	Total *n* = 241	*p* Value
Age, mean (SD)	70.17 (15.24)	62.99 (15.69)	66.77 (15.83)	0.001 **^a^
Male gender (%)	75.00 (59.10)	74.00 (64.90)	149.00 (61.80)	0.357 ^b^
Hypertension (%)	101.00 (80.20)	83.00 (72.80)	184.00 (76.70)	0.222 ^b^
Diabetes (%)	55.00 (43.70)	52.00 (45.60)	107.00 (44.60)	0.796 ^b^
Obesity (%)	26.00 (23.00)	41.00 (38.70)	67.00 (30.60)	0.013 *^b^
Hyperlipidemia (%)	76.00 (60.30)	69.00 (61.10)	145.00 (60.70)	1.000 ^b^
Smoking (%)	24.00 (21.60)	28.00 (28.00)	52.00 (24.60)	0.338 ^b^
Atrial fibrillation (%)	27.00 (21.40)	18.00 (15.80)	45.00 (18.80)	0.321 ^b^
Ischemic heart disease (%)	31.00 (24.60)	21.00 (18.60)	52.00 (21.80)	0.276 ^b^
Prior stroke (%)	27.00 (21.40)	23.00 (20.40)	50.00 (20.90)	0.874 ^b^
Anticoagulation (%)	33.00 (25.90)	37.00 (32.45)	70.00 (63.60)	0.327 ^b^

^a^ Independent samples *t*-test; ^b^ Chi-square test; * *p* < 0.05; ** *p* < 0.01.

**Table 2:** Characteristics of ICH patients.

Characteristics	Jews *n* = 127	Arabs *n* = 114	Total *n* = 241	*p* Value
mRS ≤ 2-baseline (%)	88.00 (71.00)	86.00 (80.40)	174.00 (75.30)	0.126 ^b^
Ventricular extension (%)	40.00 (32.30)	48.00 (43.20)	88.00 (37.40)	0.105 ^b^
Midline shift (%)	45.00 (36.30)	42.00 (37.80)	87.00 (37.00)	0.892 ^b^
Hematoma expansion (%)	22.00 (20.00)	12 (12.20)	35.00 (16.50)	0.139 ^b^
Surgical intervention (%)	22.00 (17.50)	26.00 (23.40)	48.00 (20.30)	0.262 ^b^
NIHSS, mean (SD)	7.05 (5.54)	7.38 (6.09)	7.22 (5.80)	0.710 ^a^
GSC, mean (SD)	13.37 (3.58)	12.83 (4.08)	13.11 (3.83)	0.155 ^c^
Systolic blood pressure—admission, mean (SD)	154.83 (31.03)	159.24 (33.75)	156.84 (32.29)	0.329 ^a^
Diastolic blood pressure—admission, mean (SD)	84.27 (19.99)	85.07 (17.66)	84.63 (18.94)	0.770 ^a^
ICH volume, mean (SD)	11.44 (17.81)	13.03 (18.08)	12.20 (17.92)	0.306 ^c^
PLT, mean (SD)	215.89 (63.93)	239.08 (77.98)	226.74 (71.64)	0.013 *^a^
Thrombocytopenia (%)	20.00 (1.60)	2.00 (1.80)	4.00 (1.70)	1.000 ^b^
INR, mean (SD)	1.15 (0.39)	1.10 (0.49)	1.12 (0.44)	0.401 ^a^
PTT, mean (SD)	30.70 (5.59)	29.90 (5.33)	30.31 (5.46)	0.295 ^a^
In hospital mortality (%)	10.00 (80.00)	8 (7.20)	18.00 (7.60)	1.000 ^b^
Discharged home (%)	33.00 (28.70)	38 (38.40)	71.00 (33.20)	0.147 ^b^
mRS > 3 at discharge (%)	97.00 (85.10)	84.00 (83.20)	181.00 (84.20)	0.712 ^b^

^b^ Chi-square test; ^a^ Independent samples *t*-test; ^c^ Mann–Whitney test; * *p* < 0.05

**Table 3:** Hemorrhage location and volume.

Characteristics	Jews	Arabs	Total	ICH Volume, Mean (SD)	*p* Value
	*n* = 127	*n* = 114	*n* = 241		
Deep (%)	71.00 (55.90)	64.00 (57.70)	135.00 (56.70)	8.55 (13.41) ^d^	0.007 **^b^
Lobar (%)	45.00 (35.40)	24.00 (21.60)	69.00 (29.00)	18.93 (21.69) ^d^	0.007 **^b^
Posterior fossa (%)	11.00 (8.70)	23.00 (20.70)	34.00 (14.30)	12.57 (20.95) ^d^	0.007 **^b^
ICH volume, mean (SD)	11.44 (17.81)	13.03 (18.08)	12.20 (17.92)		0.306 ^c^

^b^ Chi-square test; ^d^ Kruskal–Wallis test; ^c^ Mann–Whitney Test; ** *p* < 0.01.

**Table 4:** Difference in age (mean) of ICH patients (Arabs and Jews) in each hemorrhage region.

		Age Mean ± Std. Median (Range)	*p* Value
Deep Regions	Jews (*n* = 71)	71.20 ± 14.10 73 (32–98)	0.005 **^a^
	Arabs (*n* = 64)	64.10 ± 15.10 64.50 (25–100)	
Lobar Regions	Jews (*n* = 45)	69.70 ± 17.20 72 (20–94)	0.057 ^a^ 1-Sided *p* = 0.029 *
	Arabs (*n* = 24)	61.20 ± 17.90 64 (25–83)	
Posterior Fossa	Jews (*n* = 11)	65.10 ± 13.90 66 (38–87)	0.436 ^a^
	Arabs (*n* = 23)	60.80 ± 15.30 67 (21–84)	

^a^ Independent samples *t*-Test; * *p* < 0.05; ** *p* < 0.01.

## Data Availability

The data used in this research is not publicly available due to ethical restrictions that safeguard participant confidentiality and privacy.
